# Perspectives of patients and healthcare professionals on metabolic monitoring of adult prescribed second-generation antipsychotics for severe mental illness: A meta-synthesis

**DOI:** 10.1371/journal.pone.0283317

**Published:** 2023-04-19

**Authors:** Pooja Gopal Poojari, Sohil Khan, Sonia Shenoy, Sahana Shetty, Keshava Pai, Leelavathi D. Acharya, Swarnali Bose, Girish Thunga

**Affiliations:** 1 Department of Pharmacy Practice, Manipal College of Pharmaceutical Sciences, Manipal Academy of Higher Education, Manipal, Karnataka, India; 2 School of Pharmacy and Medical Sciences, Menzies Health Institute, Gold Coast, Griffith University, Southport, Australia; 3 Department of Psychiatry, Kasturba Medical College, Manipal, Manipal Academy of Higher Education, Manipal, Karnataka, India; 4 Department of Endocrinology, Kasturba Medical College, Manipal, Manipal Academy of Higher Education, Manipal, Karnataka, India; 5 Department of Psychiatry, Kasturba Medical College, Mangalore, Karnataka, India; 6 Department of Clinical Psychology, Central Institute of Psychiatry, Ranchi, Jharkhand, India; University of South Australia, AUSTRALIA

## Abstract

**Objectives:**

We conducted a meta-synthesis of qualitative studies to synthesize the views of psychiatric patients on second-generation antipsychotics (SGAs) and the healthcare providers about the metabolic monitoring of adult-prescribed SGAs.

**Methods:**

A systematic search was conducted in four databases through SCOPUS, PubMed, EMBASE, and CINAHL to identify qualitative studies of patients’ and healthcare professionals’ perspectives on the metabolic monitoring of SGAs. Initially, titles and abstracts were screened to exclude articles that were not relevant followed by full-text reading. Study quality was assessed by using Critical Appraisal Skills Program (CASP) criteria. Themes were synthesized and presented as per the Interpretive data synthesis process (Evans D, 2002).

**Results:**

A total of 15 studies met the inclusion criteria and were analyzed in meta-synthesis. Four themes were identified: 1. Barriers to metabolic monitoring; 2. Patient related concerns to metabolic monitoring; 3. Support system by mental health services to promote metabolic monitoring; and 4. Integrating physical health with mental health services. From the participants’ perspectives, barriers to metabolic monitoring were accessibility of services, lack of education and awareness, time/resource constraints, financial hardship, lack of interest on metabolic monitoring, patient capacity and motivation to maintain physical health and role confusion and impact on communication. Education and training on monitoring practices as well as integrated mental health services for metabolic monitoring to promote quality and safe use of SGAs are the most likely approaches to promote adherence to best practices and minimize treatment-related metabolic syndrome in this highly vulnerable cohort.

**Conclusion:**

This meta-synthesis highlights key barriers from the perspectives of patients and healthcare professionals regarding the metabolic monitoring of SGAs. These barriers and suggested remedial strategies are important to pilot in the clinical setting and to assess the impact of the implementation of such strategies as a component of pharmacovigilance to promote the quality use of SGAs as well as to prevent and/or manage SGAs-induced metabolic syndrome in severe and complex mental health disorders.

## Introduction

Antipsychotic drugs are commonly prescribed for various psychiatric disorders. Second-generation antipsychotics (SGAs) are preferred over first-generation antipsychotics due to the lower incidence of extrapyramidal side effects. However, SGAs are also associated with adverse effects such as weight gain and glucose intolerance, which can progress into metabolic syndrome, a cluster of disorders comprising hypertension, insulin resistance, obesity, and dyslipidaemia [1]. Metabolic syndrome comprises at least three out of five metabolic risk factors: abdominal obesity, elevated blood pressure, fasting glucose, triglycerides, or decreased high-density lipoproteins [1]. Several SGAs are known to cause metabolic syndrome including olanzapine, risperidone, and clozapine [2]. Factors that contribute to the link between mental illness and poor physical health include SGAs-related adverse effects, lack of access to health services, and poor health behaviours among patients with mental illnesses [3].

Limited evidence highlights the poor frequency of physical health screening in primary care among patients with severe mental illness (SMI) [4]. Multiple factors including lack of awareness/ education about the relation between physical and mental health, lack of attention of healthcare professionals towards patients’ physical health, and patient affordability contribute to poor frequency [5]. To reduce iatrogenic morbidity and to improve the physical health monitoring of patients on SGAs, several guidelines recommend best practices but are seldom implemented in practice [1].

A qualitative study in Australia among 20 patients diagnosed with schizophrenia or major depressive disorder supports primary healthcare providers’ role in the prevention and early identification of physical health problems [6]. Providing education based on the perspectives of patients with SMI is important to prevent the adverse metabolic sequelae as evident with SGAs [4].

In US, a qualitative study [7] conducted among patients with severe and persistent mental health disorders revealed a lack of seeking care for physical health because they were afraid of being detained for their mental health problems if they have interaction with the health system. Stigma, variation in the quality of care offered, lack of assessment and monitoring of physical health issues and need the for the expertise of health care teams to manage the complexity of issues posed by people living with an SMI are the factors that affect a person’s access to and provision of physical health care [8].

To determine how to best reform healthcare to enhance access to quality physical health care for people with mental illness, all stakeholders’ perspectives must be sought and addressed. For example, understanding nurses’ attitudes and practices when it comes to physical health treatment [3] could help strengthen the patient-practitioner interface in raising awareness. There is limited research into patients’ and healthcare professionals’ perspectives on the physical health of those with mental illness. Because people with mental illnesses are often socially isolated, carers’ participation is crucial, as they may be the only source of support that would also help in the development of integrated care techniques [9]. Increased awareness of the risks associated with SGA use is critical in this vulnerable population as low levels of knowledge are also associated with poor health-related behaviours. Hence, the promotion of literacy, self-care, and involvement of family carers is important in supporting self-care [10].

To address the overburdening of physical illness in patients with SMI, it is required to first comprehend the causes and then evaluate what can be changed, and, how this change can be implemented. Through regular monitoring, appropriate management, and proper communication of results from metabolic monitoring to relevant stakeholders could play a vital role in addressing the adverse metabolic sequelae with SGAs [11]. Qualitative research helps in understanding the perception of stakeholders in identifying challenges and developing strategies for optimal health outcomes and is seldom analyzed to assess the quality of such studies and provide implications. This meta-synthesis of qualitative studies aimed to synthesize the views of psychiatric patients on SGAs and the healthcare providers about the metabolic monitoring of adult-prescribed SGAs.

## Materials and methods

### Research design

We conducted a meta-synthesis of qualitative studies by enhancing transparency in reporting the synthesis of qualitative research (ENTREQ) guidelines (S1 Appendix).

### Search strategy and study selection criteria

We used a search method relevant to each database to conduct a systematic search in four databases: SCOPUS, PubMed, EMBASE, and CINAHL. Criteria for inclusion were studies that used qualitative research and were published in English from inception to September 2022, to locate qualitative research addressing psychiatric patients aged above 18 years prescribed with SGAs and healthcare professionals’ perspectives on metabolic monitoring of those on SGAs. Studies that used quantitative methodology, children studies, other psychiatric conditions such as depression and anxiety, or those not on physical health monitoring were excluded.

In PubMed and SCOPUS, Medical Subject Headings (MeSH) phrases were used, and in CINAHL and EMBASE, CINAHL and Emtree terms were used respectively for searching the relevant studies. Keywords related to the medication such as "antipsychotic drugs", “antipsychotics” and similar terms; diagnosis-related keywords such as “psychiatric illness”, “mental illness” and other similar keywords; keywords on physical health such as “metabolic syndrome” and “metabolic monitoring”; qualitatively related keywords such as “interviews”, “qualitative research” and other such terms and health care professional related terms were used. The Boolean term OR was used within each exposure to widen the search after which the Boolean term AND was used to combine all exposures to narrow the results (The results of the search strategy of PubMed, SCOPUS, EMBASE, and CINAHL are provided in S2–S5 Appendices respectively). An example of the search strategy is provided in Box 1. Following the collection of references and the elimination of duplicates, two researchers (the first and last author) used a two-step procedure to identify studies for the analysis. The initial stage was to screen titles and abstracts to exclude non-relevant material. The entire texts of the remaining publications were assessed in the second phase to eliminate those that: (1) were not related to physical health monitoring; (2) did not cover the perspectives of patients or healthcare professionals; (3) did not use qualitative research methods; and (4) children studies and other psychiatric conditions such as depression and anxiety.

**Box 1. Example search strategy (PubMed).**

1. ((((((((((((((((((((((((((Neuroleptics) OR (Antipsychotic Medication)) OR (Medication, Antipsychotic)) OR (Neuroleptic Agent)) OR (Agent, Neuroleptic)) OR (Neuroleptic Drug)) OR (Drug, Neuroleptic)) OR (Neuroleptic)) OR (Antipsychotic)) OR (Antipsychotic Drugs)) OR (Antipsychotics)) OR (Major Tranquilizers)) OR (Neuroleptic Agents)) OR (Tranquilizing Agents, Major)) OR (Major Tranquilizing Agents)) OR (Tranquillizing Agents, Major)) OR (Major Tranquillizing Agents)) OR (Major Tranquilizer)) OR (Tranquilizer, Major)) OR (Neuroleptic Drugs)) OR (Antipsychotic Drug)) OR (Drug, Antipsychotic)) OR (Antipsychotic Agent)) OR (Agent, Antipsychotic)) OR (Antipsychotic Effect)) OR (Effect, Antipsychotic)) OR (Antipsychotic Effects)
2. (((((((((((((((((((((Metabolic Syndromes) OR (Syndrome, Metabolic)) OR (Syndromes, Metabolic)) OR (Metabolic Syndrome X)) OR (Insulin Resistance Syndrome X)) OR (Syndrome X, Metabolic)) OR (Syndrome X, Insulin Resistance)) OR (Metabolic X Syndrome)) OR (Syndrome, Metabolic X)) OR (X Syndrome, Metabolic)) OR (Dysmetabolic Syndrome X)) OR (Syndrome X, Dysmetabolic)) OR (Reaven Syndrome X)) OR (Syndrome X, Reaven)) OR (Metabolic Cardiovascular Syndrome)) OR (Cardiovascular Syndrome, Metabolic)) OR (Cardiovascular Syndromes, Metabolic)) OR (Syndrome, Metabolic Cardiovascular)) OR (Cardiometabolic Syndrome)) OR (Cardiometabolic Syndromes)) OR (Syndrome, Cardiometabolic)) OR (Syndromes, Cardiometabolic)
3. (Psychiatrists) OR (Psychiatrist)
4. (((((((((((Personnel, Health) OR (Health Care Providers)) OR (Health Care Provider)) OR (Provider, Health Care)) OR (Healthcare Providers)) OR (Healthcare Provider)) OR (Provider, Healthcare)) OR (Healthcare Workers)) OR (Healthcare Worker)) OR (Health Care Professionals)) OR (Health Care Professional)) OR (Professional, Health Care)
5. ((((((((((((((((Mental Disorder) OR (Psychiatric Illness)) OR (Psychiatric Illnesses)) OR (Psychiatric Diseases)) OR (Psychiatric Disease)) OR (Mental Illness)) OR (Illness, Mental)) OR (Mental Illnesses)) OR (Psychiatric Disorders)) OR (Psychiatric Disorder)) OR (Behavior Disorders)) OR (Diagnosis, Psychiatric)) OR (Psychiatric Diagnosis)) OR (Mental Disorders, Severe)) OR (Mental Disorder, Severe)) OR (Severe Mental Disorder)) OR (Severe Mental Disorders)
6. Research, Qualitative
7. ((((((((((Oral History as Topic) OR (Interviews, Telephone)) OR (Interview, Telephone)) OR (Telephone Interview)) OR (Telephone Interviews)) OR (Group Interviews)) OR (Group Interview)) OR (Interview, Group)) OR (Interviews, Group)) OR (Interviewers)) OR (Interviewer)

### Assessment of article quality

To determine the worth and integrity of the data used, assessing the quality of the articles was an important part of the process. We used the Critical Appraisal Skills Program (CASP) to check the quality of the eligible articles (S6 Appendix). An article was given a rating of 1 for ‘Yes’ and 0 for ‘No’ or ‘Can’t tell’. This assessment was carried out independently by two authors (the first author and the last author) and then discussed the findings with the research team to resolve any discrepancies.

### Data analysis

#### Data extraction

Two reviewers (PP and GT) independently extracted data from the included studies using a data extraction form in excel that contained information about the author, country, the year of publication, aims, study population, data collection method, sampling approach, data analysis method, and CASP score. Any discrepancies were resolved through discussion with a third reviewer (SK) and comparison with the original data.

#### Data synthesis

As per the Interpretive data synthesis process (Evans D, 2002) [12], in this step, we familiarized ourselves with the data to identify key findings. During the process of reading, texts that were determined important for meta-synthesis were copied from the results into a separate document by the first author. A matrix was built in an Excel sheet to aid in the establishment of a framework describing the document’s content. Following this, the information was transmitted to the document to determine the key findings. For each important finding, a preliminary theme was established. The list of significant findings was combined for similar themes. The important findings from studies were grouped and classified into sub-themes based on their similarities (S7 Appendix). Sub-themes were identified based on the gathered themes. Key themes and sub-themes were again reviewed and compared by assessing the raw data, participant quotes, and analysis. To detect consistencies and inconsistencies, the themes and sub-themes were re-examined and discussed with another author (SK).

## Results

### Search results

**Fig 1:**
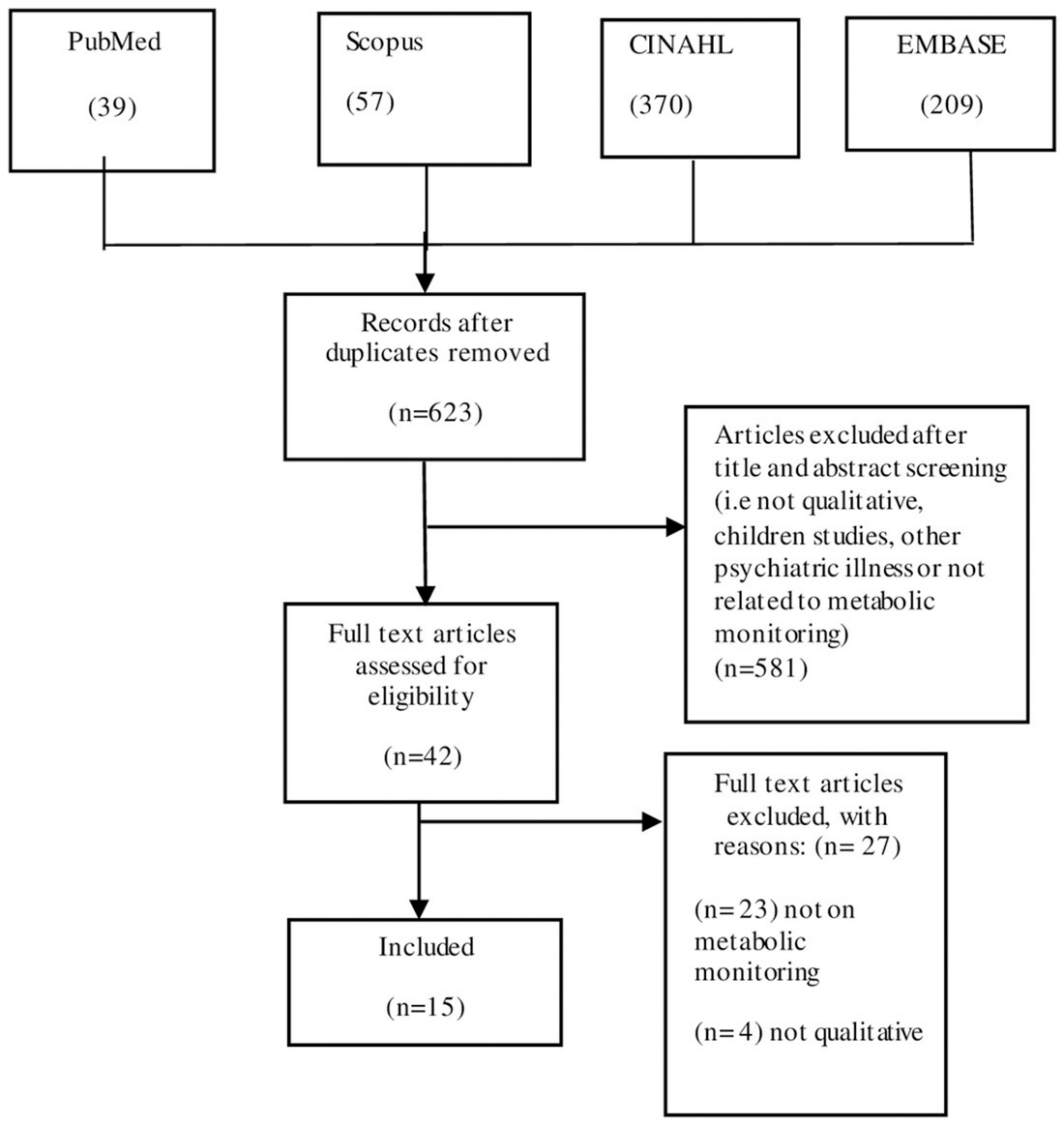
PRISMA flow diagram.

We found 39 studies in PubMed; 57 in Scopus; 209 in EMBASE and 370 in CINAHL; a total of 675 articles. After duplicates were deleted, the systematic search yielded 623 articles. Following the screening of the title and abstract, 42 full-text articles were evaluated for eligibility, among which 15 articles were included in the meta-synthesis (Fig 1).

Semi-structured or focus group interviews were used in these studies. Of the 15 papers, four addressed patients’ views only, two addressed mental health care nurses’ views only, two addressed mental health staffs’ views only, one addressed care coordinators’ views only, one addressed patients’, relatives’, and clinicians’ perspectives together, one addressed the views of community mental health team (CMHT) clinicians and patients, one addressed mental health service providers and General practitioners (GPs)’ views together and one addressed the views of patients, primary care providers, community psychiatrists, public health administrators, one addressed the views of psychiatrists, GPs, nurses, mental health pharmacists, and consumer advisors, one addressed the views of GPs, psychiatrists and mental health nurses. Six studies were from Australia, three were studies from the UK, and two studies were from Sweden, and Ireland respectively. One study from California, and New Zealand each (Table 1).

**Table 1:** Characteristics of the studies.

Author and year	Country	Aim	Population	Data collection method	Sampling approach	Data analysis method
Aouira N et al (2022) [13]	Australia	To explore the psychiatrists’ views on barriers to monitoring and strategies to improve it.	10 psychiatrists	Semi-structured interviews	Purposive sampling	Thematic analysis
Brämberg E B et al (2018) [14]	Sweden	To explore the experiences and perspectives of patients, relatives and clinicians regarding individual and organizational factors which facilitate or hinder access to somatic health care for persons with SMI.	14 patients with bipolar or schizophrenia disorder, 15 relatives and 21 clinicians	Semi-structured interviews	Strategic sampling	Content analysis
Bergqvist A et al (2013) [15]	Sweden	The purpose of this study is to explore the experiences of mental health staffs in helping mentally ill patients implement lifestyle changes to prevent metabolic syndrome	12 mental health staffs working in outpatient care	Qualitative interviews	Purposive sampling	Content analysis according to Krippendorff (2004)
Butler J (2020) [4]	UK	To understand the attitudes of CMHT clinicians and patients with SMI regarding physical healthcare	14 patients with SMI and 15 clinicians	Semi-structured interviews	Purposive sampling	Thematic analysis
Chee GL et al (2019) [16]	Australia	To understand young patients physical healthcare needs, and interest and awareness	24 young people with first episode psychosis	Semi-structured interviews	Purposeful and theoretical sampling techniques	Constant comparative method
Cocoman AM, Casey M (2018) [17]	Ireland	To determine the physical health beliefs, perceptions, and needs of community-dwelling people with mental health disorders who are taking antipsychotic medication	21 participants with SMI treated with antipsychotic medication	Focus group	Purposive sampling	Thematic research methodology (Braun & Clarke, 2006)
Collins C et al (2021) [18]	Ireland	To establish the views of Irish service providers on the physical health care of patients with SMI in order to provide information for the future development of the service	34 GPs and mental health service providers	Semi-structured interviews	Purposive sampling	Thematic analysis
Gronholm P C et al (2017) [19]	UK	This study aimed to explore care coordinators’ views and experiences regarding their ability to monitor physical health in clients with SMI	7 Care coordinators	Semi-structured interviews	Convenience sampling	Thematic analysis (Braun and Clarke)
Happell B et al (2013) [20]	Australia	To explore nurses’ views on screening/monitoring of the physical health of patients with SMI	38 Nurses from mental health care	Focus group	Convenience sampling	Thematic analysis framework (Braun and Clark)
Happell B et al (2019) [21]	Australia	The study’s main goal was to learn about patients’ perceptions of how mental health services address their physical health needs, as well as methods that have been employed or could be used to improve the current situation.	31 patients with mental illness	Focus Group	Convenience sampling	Thematic analysis using Nvivo
Mangurian C et al (2018) [22]	California (U.S)	To learn the perspectives of stakeholders on obstacles to metabolic screening for patients with SMI	8 adult patients with SMI, 8 community psychiatrists, 14 primary care providers, and 7 public health administrators	Focus group	Convenience Sampling	Dedoose®
Nankivell J et al (2013) [23]	Australia	To investigate the views of nurses within the public mental health system on the physical health, of patients with SMI	38 nurses from mental health care	Focus group	Convenience sampling	Thematic analysis (Braun and Clarke)
Nash A et al (2021) [24]	UK	To explore healthcare professionals’ views on physical health of people with SMI	9 GPs, 10 psychiatrists and 4 mental health nurses	Semi-structured interviews	Purposive sampling	Constant comparison method
Wheeler AJ et al (2010) [25]	New Zealand	To explain the findings of health practitioners’ perspectives on their involvement in assessing and managing their patients cardiovascular risk profiles.	9 interviews with key informants (psychiatrists, GPs, nurse specialists, consumer advisors and mental health pharmacist)	Semi-structured interviews	Purposive sampling	Content analysis
Young SJ et al (2017) [26]	Australia	From the patients’ perspective, investigating the role of mental health services in enhancing physical health among people with SMI.	40 patients	Semi-structured interviews	Convenience sampling	Modified framework approach (Ritchie & Spencer 1994)

### Quality assessment

Overall, the majority of studies that were evaluated with CASP showed positive results with a score of 9 (out of 10) except for two studies which scored 7 and 8 [4, 22] respectively. Author reflexivity (Q6), which is defined as a critical evaluation of the author’s own part in every phase of the research process, was occasionally insufficient or not clearly mentioned (Table 2 and S8 Appendix).

**Table 2:** Quality appraisal of the eligible studies using CASP.

Authors and Year	Q1	Q2	Q3	Q4	Q5	Q6	Q7	Q8	Q9	Q10	Total score
Aouira N et al(2022) [13]	Y	Y	Y	Y	Y	N	Y	Y	Y	Y	9
Brämberg E B et al, 2018 [14]	Y	Y	Y	Y	Y	N	Y	Y	Y	Y	9
Bergqvist A et al,2013 [15]	Y	Y	Y	Y	Y	N	Y	Y	Y	Y	9
Butler J et al(2020) [4]	Y	Y	Y	C	Y	N	Y	Y	Y	Y	8
Chee GL et al,2019 [16]	Y	Y	Y	Y	Y	C	Y	Y	Y	Y	9
Cocoman AM, Casey M, 2018 [17]	Y	Y	Y	Y	Y	N	Y	Y	Y	Y	9
Collins C et al,2021 [18]	Y	Y	Y	Y	Y	N	Y	Y	Y	Y	9
Gronholm P C et al,2017 [19]	Y	Y	Y	N	Y	Y	Y	Y	Y	Y	9
Happell B et al,2013 [20]	Y	Y	Y	Y	Y	N	Y	Y	Y	Y	9
Happell B et al,2019 [21]	Y	Y	Y	Y	Y	C	Y	Y	Y	Y	9
Mangurian C et al,2018 [22]	Y	Y	Y	C	Y	N	Y	Y	N	Y	7
Nankivell J et al,2013 [23]	Y	Y	N	Y	Y	Y	Y	Y	Y	Y	9
Nash A et al(2021) [24]	Y	Y	Y	Y	Y	Y	Y	Y	Y	Y	10
Wheeler AJ et al [25]	Y	Y	Y	Y	Y	C	Y	Y	Y	Y	9
Young SJ et al,2017 [26]	Y	Y	Y	Y	Y	Y	Y	Y	Y	Y	10

### Themes and sub-themes

Themes and subthemes arising from the analysis of the different studies are thematically provided below and summarized in Table 3 along with the participant’s quotes from the primary studies.

**Table 3:** Themes and subthemes arising from the analysis of the different studies along with quotations from participants of primary studies.

Themes	Subthemes	Quotation from participants in primary studies
Barriers to metabolic monitoring	Accessibility of services	*Administrator*: *“There’s definitely a problem with access of referring a person to a [primary care] practitioner in a timely manner”* [22] *Patient*: *“I go to somewhere you can get bulk billed*, *but that’s not always easy*, *and it’s hard to then get the continuity in some of these big GP clinics anyway”* [21]
	Lack of education and awareness	*Patients*: *“They [doctors and nurses] just treat my mental illness*… *never really explained its [potential risks of psychosis on physical health] until later when I was out of hospital*. *Then they tried to brush up a little bit but I still don’t know to what extent the illness affects me physically”* [16] *GP*: *“Maybe you don’t have good enough specialist knowledge about these groups (author*: *patients) and the problems they can have*, *what we do in principle is treat them like any other patient*. *Then you usually get*, *can get problems with communication*, *or compliance*, *and then we usually get help from a psychiatric consultant”* [14] *Mental health staff*: *“I think it is important to educate staff; there is a tendency to forget about somatic health in psychiatric care”* [15]
	Time/resource constraints	*Community mental health team*: *“it’s often difficult as a psychiatrist to devote times to these matters and follow through with people so it comes down to a reasonable conversation with people”* [18]
	Financial hardship	*Nurse*: *“One of the best things you can do for your physical health is have an ongoing relationship with your general practitioner*. *But our client group can’t afford it because it costs*. *You know*, *in this town*…*for the normal course of events it costs you money to go see your GP*. *And our clients just don’t have that money*. *It’s as simple as that Really*” [23]
	Lack of interest on metabolic monitoring	*Patient*: *“My mental health is more important than physical [health]”* [16] *‘I have been active my entire life in sports [before having psychosis]*… *don’t think I’ll have problems [related to physical health]* *Patient*: *“I’ve told them (about the side- effects) and they won’t do anything about it*. *They don’t offer any advice”* [26] *Mental health staffs*: *“They don’t always want to give samples and it is difficult to motivate them*. *They don’t really listen*. *They think it’s a bother and can’t manage it*” [15]
	Patient capacity and motivation to maintain physical health	*Care coordinators*: *“It’s the people we work with*, *which is who they are and negative symptoms of schizophrenia is lack of motivation*, *self-neglect”* [19] *Nurse*: *“Because they’re mentally ill*, *so sometimes*, *like as a normal person*, *they don’t perceive that idea that how much the physical health is affected as well*. *And they’re not voicing it and so that’s a problem as well*.*”* [23]
	Role confusion and impact on communication	*Psychiatrists*: *“If I ignore it*, *that could be a problem*. *If I prescribe something and it’s out of my scope—that could be a problem*. *And my question would be*: *Where does it end*? *Like*, *how far down the algorithm are we comfortable going*? [22] *Nurse*: *“We are getting referrals through from the GP*, *for a service that sees people with moderate to severe mental illness*, *so there’s a high likelihood that they are going to come to us*, *be assessed and be put on medication and the GPs are not even doing baselines*. *And then they are coming to us and we are not doing baselines either*. *So that’s always been a concern of mine that there’s a big part of our client’s health that is being missed”* [20]
Patient related concerns to metabolic monitoring	Adverse effects of antipsychotics	*Patient*: *“The medication I was on made me increased my appetite*… *and I found that I was getting bloated a lot*… *snacking more yeah*, *comfort food”* *‘I put on fifteen kilos in three months*, *it’s not good’* [16]
	Impact of medication on quality of life	*Patient*: *When you’re on medication*, *you’re tired*, *you got no motivation because you are tired*, *they are inter-related*. *My motivation is directly linked to the medication’* [16]
Support system by mental health services to promote metabolic monitoring	Education and training	*Patient*: *“The problem with information is that you pick up all these brochures and you look at it*, *and then it goes in the bin kind a thing*, *whereas if you’re actually talking to another human being who you respect then you tend to take more notice”* [26] *Patient*: *“They [doctor] explained the illness*, *what caused it*, *gave me [antipsychotic] medicine to take*… *I don’t remember that any other information was supplied*, *it’s better than nothing but it is not enough”* [16]
	Integrated mental health services for metabolic monitoring	*Psychiatrist*: *“For a range of reasons mental health service users often have poor physical health and often mental health services focus on the mental health component so I actually think they need to take a broader approach*. *And my whole perspective on it is that we need to be holistic in the way we treat people…not treat them independently of their physical issues*, *we also can’t treat them independently of the social issues impacting on those people”* [25] *Patient*: *“‘Yeah*, *a holistic way like the doctor talking to my folks to help me with my health problems’; ‘My mum was very supportive after talking to my case worker*… *she would check my weight every few days*, *watch what I eat”* [16]
	Strategies for adherence to guidelines on metabolic monitoring	*Nurse*: *“I think there needs to be a baseline of what we do*. *…There should be some routine measures that we’re completing*. *Particularly for identifying an issue and…providing some intervention for that person…it’s an opportunity as well for health promotion*.*”* [20]
Integrating physical health with mental health services	Timely communication with relevant practitioners	*Community mental health teams*: *““If we identify a problem*, *such as high cholesterol or a thyroid problem*, *we will often liaise directly with the GP by telephone or letter and ask the patient to go in to see their GP to see if they need medication… People really do need to see their GP”* [18]
	Physical-mental health prevention strategies	*Mental health staffs*: *“Ask how they live their lives*, *you receive information about their habits*, *both food habits and exercise habits*, *and how they manage their lives”* [15] *Patient*: *“‘I didn’t know I needed to change my lifestyle*, *no one told me*… *I didn’t [have to] worry about not being healthy and not feeling in shape before taking the [antipsychotic] medicine”* [16]
	Adherence to best practice guidelines	*Community mental health teams*: *“The consultant is very clued in with the NICE guidelines and from a nurse’s point of view*, *we do annual health screening for all of our clients”* [18]

#### Barriers to metabolic monitoring

This theme provides an insight into barriers in the metabolic monitoring experienced by the key stakeholders and the patients such as inaccessibility to primary health services, lack of education and awareness, time and resource constraints, financial burden, lack of interest on metabolic monitoring, patient capacity and motivation to maintain physical health and role confusion.

*Accessibility of services*. Some patients described their visits to the doctor as a favourable experience while some with SMI find it challenging to access the primary care system. Some patients had established GPs, and others found the process of accessing them difficult [21], with an administrator, providers, and nurses also acknowledging the problem [22, 23]. Patients said they were overwhelmed by the variety of healthcare alternatives available and that any assistance in choosing physicians and scheduling appointments would be immensely useful [22].

Many patients expressed feeling forced due to financial constraints, to use a large primary care facility to gain free access. However, even this type of free access was not always easy to get. Long waiting time was one of the concerns of the patients [17, 21].

Distance and transportation become even more of a problem when the patient has fewer options about where they will see a GP due to cost concerns [4], which restrict them to bulk-billing GPs according to the nurses [23].

*Lack of education and awareness*. Although most practitioners were aware of the risks of SGAs, one of the primary care participants reported that this knowledge may be restricted to secondary care because the antipsychotic medication is handled there [25].

Key respondents believed that mental health patients’ levels of comprehension and attitudes concerning their cardiovascular risk varied [4], due to various factors such as their condition, lifestyle, or sociodemographics [25]. Some patients visited the GP only when they had some serious health conditions as they attribute most of their physical symptoms to mental illness [17, 18, 25]. However, GPs and mental health experts also believed that their patients’ physical health knowledge was generally inadequate as they generally focused more on their mental health than physical health [18, 24]. One healthcare professional stated that he was unaware of the metabolic risk profile of different antipsychotics [24].

Clinicians engaged in somatic health care reported that their lack of expertise in psychiatric illness was a barrier (primary and specialized in-patient care). Their medical education included psychiatry, but this was deemed inadequate. The professionals also acknowledged that coping with noncompliance to treatment in this patient group was tough and identified the need for training to handle these issues [14, 25].

Some patients were referred to occupational therapists, physiotherapists, exercise physiologists, and dieticians after having difficulty approaching GPs and gaining recommendations for specialized services to address a variety of physical health requirements. But this was frequently after being unaware for a long period of available healthcare providers [21]. Patients expressed their belief that mental health providers prioritized mental health issues over physical health [14].

Although GPs play an important role in enhancing the physical health of patients with mental illnesses, some patients expressed concern that GPs lack enough mental health training [17, 21].

Patients would prefer to visit one doctor that could include a variety of services and felt that psychiatrists could deliver with education and training [13, 22].

Also, the patients wished that to reach a more collaborative agreement, practitioners needed to have a broader understanding of both physical and mental health [21].

However, psychiatrists also mentioned that due to a lack of expertise they did not feel confident [13] and also wished for more education on physical health [13, 15]. Some of the nurses also expressed that screening and monitoring to be incorporated into the culture (for example, by training) was thought to be difficult because of economic sources [20].

*Time/resource constraints*. Short consultations with GP had a negative influence on communication which was acknowledged by the patient group [17, 21]. When trying to educate and encourage patients to pay attention to their physical health, time restrictions in consultations were the most common challenge for GPs and psychiatrists [13, 14, 18, 24, 25]. Similar time constraints were also told by nurses [20].

Along with the difficulty in navigating the intranet system, caseloads exceeded prescribed limits, according to the care coordinators, and meeting durations were cut short due to the more number of clients to see [19]. Lack of resources for monitoring was also reported by the community mental health team clinicians and psychiatrists [4, 13, 18, 25]. As a result, the amount of work that could be accomplished during meetings was hampered and physical health monitoring and interventions were frequently pushed aside in favour of other priorities [19].

*Financial hardship*. Distance and transportation become even more of a problem when the patient has fewer options about where they will see a GP due to financial concerns that limit them to bulk-billing GPs [23]. Nurses and psychiatrists stated that most persons with SMI were not able to take care of their physical health because they cannot afford it [23, 25]. Similar financial difficulties for carers were also reported by psychiatrists [13].

*Lack of interest on metabolic monitoring*. The patients’ disinterest to undergo physical health monitoring was described by healthcare professionals and patients [13, 15, 18]. The mental health staff and GPs described patients refusing to give blood samples and also factors like forgetting to attend the follow-up [15, 18]. They concentrated on their mental disorder after knowing their diagnosis; they feel they require medical attention and assistance [16]. The CMHT also stated that the influence of their health on their socioeconomic status was viewed as a source of the risk factors experienced by patients [18]. The patients stated that their physical health is not as important as their mental health and feel that they are physically active [16, 24].

Considering the serious challenges, the psychiatrists noted that the priority for metabolic monitoring and care is diminishing [22]. The administrators also admitted that the lack of remuneration for metabolic monitoring had an impact on screening prioritization [22]. Some of the patients questioned their own ability to effectively convey their concerns to the psychiatrists [26]. A clinician also agreed that they underestimated patients’ symptoms due to the difficulty in obtaining their medical history [14].

Some patients and nurses also claimed that mental health practitioners refused to address physical health concerns when they requested them [16, 23, 26]. Whereas one psychiatrist stated that he considered changing antipsychotics only when patients raised concerns about their physical health or medicine side effects [24].

*Patient capacity and motivation to maintain physical health*. The healthcare professionals stated that they had trouble managing their patients because of issues like compliance with advice and medications, difficulty in communication, poor follow-up, and the fact that patients with SMI rarely seek physical health care more than those who do not have mental health issues [18]. Hence, psychiatrists do not interact much with patients’ lower functioning states [16, 22].

Clients’ definitions of health, which often differed from those of healthcare professionals, were also impacted by psychotic symptoms, according to care coordinators. This discrepancy may act as a deterrent to physical health interventions [19].

Low motivation had become a way of life for patients, reducing their willingness to increase physical activity and adopt good living habits [15, 19]. Individuals linked their mental health to their desire and capacity to care for their physical health; the better their mental health, the more chance to engage in care behaviour [23, 26]. Hence, their cognitive disabilities were the main barriers to accessing physical health according to the patients and clinicians [14].

*Role confusion and impact on communication*. Practitioners’ comments also highlighted on lack of clarity regarding who is responsible for monitoring and challenges with following up patients with SMI [13, 20, 24, 25].

Psychiatrists had mixed views on broadening their area of practice. Some psychiatrists agreed given the negative effects of antipsychotic drugs, providing treatment for various metabolic problems should be within their scope of practice [13]. Contrary to belief, monitoring, management, and follow-up of the known risks were regarded as outside the scope of mental health services by some psychiatrists [4, 22, 25, 26]. According to the care coordinators, psychiatrists and government regulations expected GPs to take care of patients’ physical health [19].

Some healthcare professionals, however, viewed responsibility for diagnosing and managing physical well-being as a joint responsibility between primary and secondary care [13, 25]. Patients also were confused regarding the role of who should be responsible for their physical health monitoring [4].

Generally, the collaboration between GPs and CMH teams was seen as non-systematic and less-than-ideal [18]. The lack of coordination between different healthcare systems was another barrier reported by patients and clinicians [13, 14, 25].

Patients were employed as messengers to transfer information, according to GPs and CMH team members, who did not directly get information from the other party. This was deemed particularly worrisome due to the patient’s potential inability to recall important knowledge [18]. Poor communication was mentioned by GP, nurses, and CMH team members as a reason for blood tests, being performed by the GP only weeks after the consultant psychiatrist had performed them. For the patients, this was a source of frustration [18].

#### Patient related concerns to metabolic monitoring

SGAs have been linked to an increase in risk factors for metabolic syndrome in mental health patients. Concerns about these medications’ side effects were prominent, owing to prescribing instructions that favoured second-generation treatments.

*Adverse effects of antipsychotics*. The most common concerns expressed by the patients were weight and/or physical shape which made them feel embarrassed and harmed their confidence [17, 26]. Other patients spoke about the long-term side effects of the medication and their effects on their bodies such as twitches, dry mouth, and sweating [17]. They stated that when they first started taking antipsychotic drugs, they were given very little information regarding the prescriptions and the potential side effects [16].

Furthermore, the healthcare professionals mentioned that the patients’ appetites had increased and the medication’s side effects frequently included metabolic abnormalities such as weight gain, high blood pressure, and type 2 diabetes [15, 16, 25].

Nurses stated that antipsychotics’ significant side effects necessitated additional screening to create baselines for monitoring and addressing adverse effects [20].

*Impact of medication on quality of life*. Although few patients considered themselves to be healthy, the majority expressed various health-related worries, expressing a variety of negative effects on daily activities, social interaction, and quality of life.

Antipsychotic medicines made patients feel drowsy, making it difficult to engage in physical activity, according to the care coordinators [19]. Patients stated that they had no motivation even to carry out their daily activities, affecting their self-esteem [16].

The combined effects of antipsychotic drugs and psychotic symptoms on clients’ motivation to engage in healthy lifestyles, as well as the consequences for their physical health were noted by care coordinators [19].

#### Support system by mental health services to promote metabolic monitoring

The majority of the participants expect that doctors and mental health services should also provide additional lifestyle-related education along with regular monitoring. They frequently stated that they expected physicians to communicate more effectively with others involved in their care to improve outcomes. The secondary care providers also stressed the importance of taking a holistic approach to treatment.

*Education and training*. While some patients preferred printed materials, others thought an interactive approach would be more relevant and beneficial [26].

Patients expressed their disappointment that they were not given adequate opportunities to learn how to care for their physical health [16].

However, patients indicated health awareness, nutritional advice, and knowledge of their condition helped to mitigate the negative effects of the symptoms they were facing [16, 17].

One patient expressed a wish to collaborate with a team to discuss drug choices but was not supported by their practitioner [21]. Stigma can be addressed through education and experience, and one nurse mentioned that such programs for GPs improved both the level of health service offered to persons with SMI and the experience for the GPs [23].

*Integrated mental health services for metabolic monitoring*. From personal experience, one patient discussed the importance of collaboration among psychiatrists, psychologists, GPs, caseworkers, and naturopaths in improving one’s physical health prospects [21]. A similar statement was made by patients and clinicians, where they felt that a holistic approach would help in achieving positive outcomes related to their physical health [4, 16, 24, 26].

Psychiatrists’ roles have been defined as being centred on their client’s mental health, but they have evolved to include social and physical well-being as well, rather than a narrow focus on mental health in isolation according to a care coordinator and psychiatrists [19, 25].

Physical and mental health are intertwined, according to the patients, and paying attention to physical health is crucial in improving mental health outcomes [17], which has been neglected most of the time, focusing only on mental health conditions [21].

GPs, according to nurses, did not take a holistic perspective of a patient’s health, instead, segmenting it into mental and physical health [23].

Integrated communication, according to the GPs and care coordinators, is a potential technique for working with SMI patients [18, 19].

*Strategies for adherence to guidelines on metabolic monitoring*. Some community mental health teams informed that for all of their attending patients with SMI, a full annual physical health examination was in place, according to the National Institute for Health and Care Excellence (NICE) criteria [18]. Some of the patients also expressed dissatisfaction that they have not been followed up regarding their chronic conditions or side effects of the medication [26].

Nurses believed that screening should be routine in general and noted a variety of clinical and broader benefits, such as offering an opportunity to communicate with patients about health promotion.

While emphasizing the need for baselines in any scenario, nurses also indicated that antipsychotic-related clinical problems could emerge over time, therefore screening is essential and necessary at regular intervals [20].

Annual reminders about primary health care and mental health outpatient consultations were regarded as organizational facilitators by both patients and clinicians. The clinicians found that the annual health surveys for psychiatric patients provided them with more information and made it easier to communicate with them [14].

#### Integrating physical health with mental health services

This theme describes the effective management ideas about their physical health followed by the healthcare professionals towards their patients. While a few patients said they did not remember being questioned about physical health, some of the participants remembered being asked about their lifestyle, referral to the GP, as well as having their blood pressure, and weight measured and blood sample checks.

*Timely communication with relevant practitioners*. Some patients also expressed how their physical health needs were satisfied, as well as how the mental health treatment assisted them in improving their physical health and general well-being [14, 17]. Community mental health teams reported that in case of any physical health problem they refer to their GP [18]. If necessary, the physician prescribed lipid-lowering medicine or sent a referral letter to the district health care facility [15]. One of the patients stated that their routine tests were supported by the nurse [21].

*Physical-mental health prevention strategies*. Lifestyle modifications were suggested by most psychiatrists and other staff for metabolic disturbances [15, 24]. Patients also mentioned the help they had received from the mental health service’s self-management classes [15, 17]. Some healthcare professionals looked into nutrition, exercise, smoking habits, and alcohol intake as well as other health issues. In certain circumstances, patients were told to keep a food journal in which they may note their diet schedule. Those who had not been exercising frequently before being diagnosed with psychosis were advised to go for a 10-minute walk every day [15]. However, one of the patients told of being unaware of lifestyle modifications [16]. But as changing one’s lifestyle was an individual preference, also the side effects of SGAs caused difficulties in making lifestyle modifications, and doctors’ influence on behaviour modification was limited according to the key informants [15, 25]. Some of the psychiatrists also reported changing higher-risk antipsychotic drugs to drugs with lower metabolic risk [24].

*Adherence to best practice guidelines*. During treatment with SGA medications, some of the individuals underwent weight and blood sample checks, according to healthcare professionals, but this was not always possible due to resource and time constraints [4, 13, 15, 18]. Also, the nurses and key respondents stated that despite the procedures in existence and the increase in monitoring from a few years, basic checks, such as those required to track changes in clinical parameters, were not being performed regularly due to the discrepancies between the guidelines and practice in screening [20, 25].

Healthcare professionals also discussed at the treatment conference about the weight issue and the need to motivate the patients to go for monitoring [15].

## Discussion

We aimed to synthesize the views of healthcare professionals and patients diagnosed with psychiatric conditions who were on antipsychotics, and their experience with the metabolic monitoring of SGAs through a thematic approach; and identified key barriers and strategies to adhere to metabolic monitoring parameters as one of the evidence-based approaches to promote quality use of SGAs. Previous reports have shown that metabolic adverse effects of SGAs are a major concern in patients prescribed SGA drugs.

Lack of motivation, psychotic symptoms, a lack of understanding of health-related matters, and medication side effects was among them (weight gain mainly), all of these criteria appear to be crucial, and they appear to make them choose a healthy lifestyle incredibly difficult.

Our findings suggest that some patients with SMI have good, mutually beneficial relationships with their GPs and psychiatrists as some of the patients had previously established doctors who were easily accessible to them. Even among those who were able to explore the system, some people are frustrated by the necessity to see multiple doctors for various medical issues. According to the data relevant to the first theme, one of the reasons for looking for a variety of providers is the difficulty in meeting GPs as their initial point of contact in seeking physical treatment. Others are far too mentally unwell to manage their own care and providers noted that metabolic monitoring rarely was the primary intention of their healthcare visits.

Even though the financial burden faced by many patients, which creates a hurdle in accessing primary care services, was another barrier mentioned by key informants, it was not much highlighted in our study as only three studies supported this sub-theme.

A cross-sectional survey conducted by Abdulhaq B showed that the financial issues and lack of compliance by the family and patients as barriers to metabolic screening [27]. However, healthcare professionals in our study recognized that their client’s ability to take healthy actions is also hampered by mental symptoms, poor cognitive functioning, physical health issues, and a lack of health information. Similarly, the patient-related barriers to monitoring and management of metabolic health identified by a systematic review conducted by Ali RA et al were disability due to their condition, lack of knowledge, and skills [28]. More research is needed to better understand what individuals with major mental illness know about health risks and healthy lifestyle behaviour.

SGAs, according to the majority of key informants, are linked to an increased incidence of physical health side effects, which could have a significant influence on cardiovascular risk. Because the GPs had insufficient knowledge and experience with these drugs, the key informants’ views on antipsychotics varied. Identifying who is responsible for risk assessment, management, and follow-up prompted concerns regarding role confusion and boundary difficulties. Some people preferred that primary care take responsibility, while others suggested that improving CVD outcomes should be a joint responsibility between the psychiatrist and GP. As in our study, GPs believed this was their job in persons with SMI, but a limitation of knowledge of antipsychotics’ CVD-related side effects was cited as a barrier. A survey conducted by Mangurian C of 214 primary care providers showed that around 40% of them were unaware of the guidelines for metabolic monitoring and variation was observed in the responses about the responsibility of monitoring patients’ metabolic issues [29]. To ensure timely access to preventive care and treatment, mental health services and GPs must clarify responsibilities at the system and local levels, as clarifying the roles of health care professionals to be in-charge of physical health can improve monitoring [26]. Clinicians must be trained in the use of existing practice guidelines for monitoring and managing common physical conditions among people with SMI and they must have the tools and infrastructure to put these guidelines into practice [30].

Patients regularly discussed weight gain with medicines, but they did not mention routine screening or metabolic health awareness. Although a number of them had used drugs that led them to gain weight, no patient reported receiving regular metabolic monitoring. A retrospective cohort study conducted by Poojari PG et al in South India, showed a low rate of documentation for metabolic parameters in these patients [31]. Patients who use antipsychotic drugs often acquire major physical issues as a result of their treatment, or they become so drowsy and overweight that they are unable to leave their homes, or engage in meaningful social interaction or regular physical activity.

Patients also complained about a lack of information from doctors regarding the antipsychotic medications they were taking, the need of maintaining physical health, and the importance of making good lifestyle choices. A qualitative interview conducted by Hardy S in 2012 with five patients to understand their views on physical health showed that they would like to have more information about blood tests, medication, and healthy lifestyles [32]. Increased awareness of health can help patients make better lifestyle changes and gain confidence in their ability to work collaboratively with healthcare professionals. However, the healthcare professionals stated that motivating patients to make lifestyle changes is a continuous encouragement process that must be performed and reviewed regularly. Even if the majority of people with psychotic disorders understood the value of having a healthy lifestyle, negative symptoms (alogia, avolition) and cognitive impairments (problems remembering or understanding) hampered their ability to adopt healthy lifestyle behaviours.

### Strengths and limitations

To our best knowledge, this is the first meta-synthesis that provides comprehensive information on the factors that, according to the patients, psychiatrists, nurses, and GPs, can hinder physical health monitoring in patients on SGAs. By synthesizing the views identified by these key stakeholders, this paper can help in the analyses of barriers to metabolic monitoring and offer strategies to implement them more effectively in practice.

This review is limited to primary studies published in the English language only due to limited access to translating services. Besides, the authors were familiar with English language study reviews. Another limitation is, even though authors have tried their best to exclude the studies on depression and anxiety while first-pass and second-pass screening, however, some studies included in this review did not provide information on the diagnosis of patient condition.

## Implications and conclusion

Through this meta-synthesis, we described the perspectives of psychiatric patients and their healthcare providers on the metabolic monitoring of SGAs. Raising patient understanding and knowledge of their physical health issues is the first step toward increasing participation in regular metabolic monitoring. Importantly, other potential stakeholders, including caregivers and policymakers, should be explored through future qualitative research to gain better insight at the community and healthcare system level. The key barriers identified and suggested remedial strategies are important to pilot in the clinical setting and to assess the impact of the implementation of such strategies as a component of pharmacovigilance to promote quality use of SGAs as well as to prevent and/or manage SGAs-induced metabolic syndrome in severe and complex mental health disorders.

## Supporting information

S1 Appendix The ENTREQ checklist. (PDF)

S2 Appendix PubMed search history results. (CSV)

S3 Appendix SCOPUS search history results. (PDF)

S4 Appendix EMBASE search history results. (CSV)

S5 Appendix CINAHL search history results. (PDF)

S6 Appendix CASP checklist. (PDF)

S7 Appendix Final articles screened obtained after duplicates removal, key findings, themes, sub-themes and participant quotes. (XLSX)

S8 Appendix CASP scores. (XLSX)
