# Supplementary material for: Perspectives of patients and healthcare professionals on metabolic monitoring of adult prescribed second-generation antipsychotics for severe mental illness: A meta-synthesis
Source: PLoS One. 2023 Apr 19;18(4):e0283317. doi: 10.1371/journal.pone.0283317 (PMC10115273; doi:10.1371/journal.pone.0283317)
Supplement: S3 Appendix — (PDF) [file pone.0283317.s003.pdf]

Start exploring

Discover the most reliable, relevant, up-to-date research. All in one place.

Documents | Authors | Researcher Discovery *Pilot* | Affiliations

Search tips

Search within
All fields

Search documents *
"Oral History as Topic"

OR

Search within
All fields

Search documents
"Interviews, Telephone"

OR

Search within
All fields

Search documents
"Interview, Telephone"

OR

Search within
All fields

Search documents
"Telephone Interview"

OR

Search within
All fields

Search documents
"Telephone Interviews"

OR

Search within
All fields

Search documents
"Group Interviews"

OR

Search within
All fields

Search documents
"Group Interview"

OR

Search within
All fields

Search documents
"Interview, Group"

OR

Search within
All fields

Search documents
"Interviews, Group"

OR

Search within
All fields

Search documents
Interviewers

OR

Search within
All fields

Search documents
Interviewer

+ Add search field | Add date range | Advanced document search >

Reset | Search

Search History | Saved Searches

Combine queries

13 ((ALL (neuroleptics) OR ALL ("antipsychotic medication") OR ALL ("medication, antipsychotic") OR ALL ("neuroleptic agent") OR ALL ("agent, neuroleptic") OR ALL ("neuroleptic drug") OR ALL ("drug, neuroleptic") OR ALL (neuroleptic) OR ALL (neuroleptic) OR ALL (antipsychotic) OR ALL ("antipsychotic drugs") OR ALL (antipsychotics) OR ALL ("major tranquilizers") OR ALL ("neuroleptic agents") OR ALL ("tranquilizing agents, major") OR ALL ("major tranquilizing agents") OR ALL ("tranquillizing agents, major") OR ALL ("major tranquillizing agents") OR ALL ("major tranquilizer") OR ALL ("tranquilizer, major") OR ALL ("neuroleptic drugs") OR ALL ("antipsychotic drug") OR ALL ("drug, antipsychotic") OR ALL ("antipsychotic agent") OR ALL ("agent, antipsychotic") OR ALL ("antipsychotic effect") OR ALL ("effect, antipsychotic") OR ALL ("antipsychotic effects"))) AND ((ALL ("metabolic syndromes") OR ALL ("syndrome, metabolic") OR ALL ("syndromes, metabolic") OR ALL ("metabolic syndrome x") OR ALL ("insulin resistance syndrome x") OR ALL ("syndrome x, metabolic") OR ALL ("syndrome x, insulin resistance") OR ALL ("metabolic x syndrome") OR ALL ("syndrome, metabolic x") OR ALL ("x syndrome, metabolic") OR ALL ("dysmetabolic syndrome x") OR ALL ("syndrome x, dysmetabolic") OR ALL ("reaven syndrome x") OR ALL ("syndrome x, reaven") OR ALL ("metabolic cardiovascular syndrome") OR ALL ("cardiovascular syndrome, metabolic") OR ALL ("cardiovascular syndromes, metabolic") OR ALL ("syndrome, metabolic cardiovascular") OR ALL ("cardiometabolic syndrome") OR ALL ("cardiometabolic syndromes") OR ALL ("syndrome, cardiometabolic") OR ALL ("syndromes, cardiometabolic"))) AND (((ALL ("personnel, health") OR ALL ("health care providers") OR ALL ("health care provider") OR ALL ("provider, health care") OR ALL ("healthcare providers") OR ALL ("healthcare provider") OR ALL ("provider, healthcare") OR ALL ("healthcare workers") OR ALL ("healthcare worker") OR ALL ("health care professionals") OR ALL ("health care professional") OR ALL ("professional, health care"))) OR ((ALL ("mental disorder") OR ALL ("psychiatric illness") OR ALL ("psychiatric illnesses") OR ALL ("psychiatric diseases") OR ALL ("psychiatric disease") OR ALL ("mental illness") OR ALL ("illness, mental") OR ALL ("mental illnesses") OR ALL ("psychiatric disorders") OR ALL ("psychiatric disorder") OR ALL ("behavior disorders") OR ALL ("diagnosis, psychiatric") OR ALL ("psychiatric diagnosis") OR ALL ("mental disorders, severe") OR ALL ("mental disorder, severe") OR ALL ("severe mental disorder") OR ALL ("severe mental disorders"))) OR ((ALL (psychiatrists) OR ALL (psychiatrist)))) AND ((ALL ("research, qualitative")) OR ((ALL ("oral history as topic") OR ALL ("interviews, telephone") OR ALL ("interview, telephone") OR ALL ("telephone interview") OR ALL ("telephone interviews") OR ALL ("group interviews") OR ALL ("group interview") OR ALL ("interview, group") OR ALL ("interviews, group") OR ALL (interviewers) OR ALL (interviewer))))

57 results | Set Alert | More

Show less

12 (ALL ("research, qualitative")) OR ((ALL ("oral history as topic") OR ALL ("interviews, telephone") OR ALL ("interview, telephone") OR ALL ("telephone interview") OR ALL ("telephone interviews") OR ALL ("group interviews") OR ALL ("group interview") OR ALL ("interview, group") OR ALL ("interviews, group") OR ALL (interviewers) OR ALL (interviewer)))

117,598 results | Set Alert | More

Show less

11 ((ALL ("personnel, health") OR ALL ("health care providers") OR ALL ("health care provider") OR ALL ("provider, health care") OR ALL ("healthcare providers") OR ALL ("healthcare provider") OR ALL ("provider, healthcare") OR ALL ("healthcare workers") OR ALL ("healthcare worker") OR ALL ("health care professionals") OR ALL ("health care professional") OR ALL ("professional, health care"))) OR ((ALL ("mental disorder") OR ALL ("psychiatric illness") OR ALL ("psychiatric illnesses") OR ALL ("psychiatric diseases") OR ALL ("psychiatric disease") OR ALL ("mental illness") OR ALL ("illness, mental") OR ALL ("mental illnesses") OR ALL ("psychiatric disorders") OR ALL ("psychiatric disorder") OR ALL ("behavior disorders") OR ALL ("diagnosis, psychiatric") OR ALL ("psychiatric diagnosis") OR

1,509,850 results | Set Alert | More

ALL ("mental disorders, severe") OR ALL ("mental disorder, severe") OR ALL ("severe mental disorder") OR ALL ("severe mental disorders"))) OR ((ALL (psychiatrists) OR ALL (psychiatrist)))

Show less

7	(ALL ("oral history as topic") OR ALL ("interviews, telephone") OR ALL ("interview, telephone") OR ALL ("telephone interview") OR ALL ("telephone interviews") OR ALL ("group interviews") OR ALL ("group interview") OR ALL ("interview, group") OR ALL ("interviews, group") OR ALL (interviewers) OR ALL (interviewer)) Show less	110,754 results Set Alert More
6	ALL ("research, qualitative")	7,184 results Set Alert More
5	(ALL (psychiatrists) OR ALL (psychiatrist))	274,637 results Set Alert More
4	(ALL ("mental disorder") OR ALL ("psychiatric illness") OR ALL ("psychiatric illnesses") OR ALL ("psychiatric diseases") OR ALL ("psychiatric disease") OR ALL ("mental illness") OR ALL ("illness, mental") OR ALL ("mental illnesses") OR ALL ("psychiatric disorders") OR ALL ("psychiatric disorder") OR ALL ("behavior disorders") OR ALL ("diagnosis, psychiatric") OR ALL ("psychiatric diagnosis") OR ALL ("mental disorders, severe") OR ALL ("mental disorder, severe") OR ALL ("severe mental disorder") OR ALL ("severe mental disorders")) Show less	1,089,729 results Set Alert More
3	(ALL ("personnel, health") OR ALL ("health care providers") OR ALL ("health care provider") OR ALL ("provider, health care") OR ALL ("healthcare providers") OR ALL ("healthcare provider") OR ALL ("provider, healthcare") OR ALL ("healthcare workers") OR ALL ("healthcare worker") OR ALL ("health care professionals") OR ALL ("health care professional") OR ALL ("professional, health care")) Show less	332,172 results Set Alert More
2	(ALL ("metabolic syndromes") OR ALL ("syndrome, metabolic") OR ALL ("syndromes, metabolic") OR ALL ("metabolic syndrome x") OR ALL ("insulin resistance syndrome x") OR ALL ("syndrome x, metabolic") OR ALL ("syndrome x, insulin resistance") OR ALL ("metabolic x syndrome") OR ALL ("syndrome, metabolic x") OR ALL ("x syndrome, metabolic") OR ALL ("dysmetabolic syndrome x") OR ALL ("syndrome x, dysmetabolic") OR ALL ("reaven syndrome x") OR ALL ("syndrome x, reaven") OR ALL ("metabolic cardiovascular syndrome") OR ALL ("cardiovascular syndrome, metabolic") OR ALL ("cardiovascular syndromes, metabolic") OR ALL ("syndrome, metabolic cardiovascular") OR ALL ("cardiometabolic syndrome") OR ALL ("cardiometabolic syndromes") OR ALL ("syndrome, cardiometabolic") OR ALL ("syndromes, cardiometabolic")) Show less	477,381 results Set Alert More
1	(ALL (neuroleptics) OR ALL ("antipsychotic medication") OR ALL ("medication, antipsychotic") OR ALL ("neuroleptic agent") OR ALL ("agent, neuroleptic") OR ALL ("neuroleptic drug") OR ALL ("drug, neuroleptic") OR ALL (neuroleptic) OR ALL (neuroleptic) OR ALL (antipsychotic) OR ALL ("antipsychotic drugs") OR ALL (antipsychotics) OR ALL ("major tranquilizers") OR ALL ("neuroleptic agents") OR ALL ("tranquilizing agents, major") OR ALL ("major tranquilizing agents") OR ALL ("tranquillizing agents, major") OR ALL ("major tranquillizing agents") OR ALL ("major tranquilizer") OR ALL ("tranquilizer, major") OR ALL ("neuroleptic drugs") OR ALL ("antipsychotic drug") OR ALL ("drug, antipsychotic") OR ALL ("antipsychotic agent") OR ALL ("agent, antipsychotic") OR ALL ("antipsychotic effect") OR ALL ("effect, antipsychotic") OR ALL ("antipsychotic effects")) Show less	278,965 results Set Alert More
