# Supplementary material for: Perspectives of patients and healthcare professionals on metabolic monitoring of adult prescribed second-generation antipsychotics for severe mental illness: A meta-synthesis
Source: PLoS One. 2023 Apr 19;18(4):e0283317. doi: 10.1371/journal.pone.0283317 (PMC10115273; doi:10.1371/journal.pone.0283317)
Supplement: S5 Appendix — (PDF) [file pone.0283317.s005.pdf]

#	Query	Limiters/Expanders	Results
S5	S1 AND S2 AND S3 AND S4	Expanders - Apply equivalent subjects<br>Search modes - Boolean/Phrase	370
S4	TX qualitative research or qualitative study or qualitative methods or interview	Expanders - Apply equivalent subjects<br>Search modes - Boolean/Phrase	583,703
S3	TX antipsychotics or antipsychotic medication or antipsychotic drug or psychotropic	Expanders - Apply equivalent subjects<br>Search modes - Boolean/Phrase	51,276
S2	TX (psychiatrists or mental health professionals) OR TX (healthcare professionals or healthcare workers or healthcare providers or physician or nurse or doctor) OR TX (psychiatric patients or mental health patients or behavioral health patients)	Expanders - Apply equivalent subjects<br>Search modes - Boolean/Phrase	1,805,515
S1	TX metabolic syndrome or metabolic disease or syndrome x	Expanders - Apply equivalent subjects<br>Search modes - Boolean/Phrase	55,613
